# AI Interventions to Alleviate Healthcare Shortages and Enhance Work Conditions in Critical Care: Qualitative Analysis

**DOI:** 10.2196/50852

**Published:** 2025-01-13

**Authors:** Nadine Bienefeld, Emanuela Keller, Gudela Grote

**Affiliations:** 1 ETH Zurich Zurich Switzerland; 2 University Hospital of Zurich Zurich Switzerland

**Keywords:** artificial intelligence, AI, work design, sociotechnical system, work, job, occupational health, sociotechnical, new work, future of work, satisfaction, health care professionals, intensive care, ICU, stress mitigation, worker, employee, stress, health care professional, overburdened, burden, burnout, autonomy, competence, flexible, task, workplace, hospital

## Abstract

**Background:**

The escalating global scarcity of skilled health care professionals is a critical concern, further exacerbated by rising stress levels and clinician burnout rates. Artificial intelligence (AI) has surfaced as a potential resource to alleviate these challenges. Nevertheless, it is not taken for granted that AI will inevitably augment human performance, as ill-designed systems may inadvertently impose new burdens on health care workers, and implementation may be challenging. An in-depth understanding of how AI can effectively enhance rather than impair work conditions is therefore needed.

**Objective:**

This research investigates the efficacy of AI in alleviating stress and enriching work conditions, using intensive care units (ICUs) as a case study. Through a sociotechnical system lens, we delineate how AI systems, tasks, and responsibilities of ICU nurses and physicians can be co-designed to foster motivating, resilient, and health-promoting work.

**Methods:**

We use the sociotechnical system framework COMPASS (Complementary Analysis of Sociotechnical Systems) to assess 5 job characteristics: autonomy, skill diversity, flexibility, problem-solving opportunities, and task variety. The qualitative analysis is underpinned by extensive workplace observation in 6 ICUs (approximately 559 nurses and physicians), structured interviews with work unit leaders (n=12), and a comparative analysis of data science experts’ and clinicians’ evaluation of the optimal levels of human-AI teaming.

**Results:**

The results indicate that AI holds the potential to positively impact work conditions for ICU nurses and physicians in four key areas. First, autonomy is vital for stress reduction, motivation, and performance improvement. AI systems that ensure transparency, predictability, and human control can reinforce or amplify autonomy. Second, AI can encourage skill diversity and competence development, thus empowering clinicians to broaden their skills, increase the polyvalence of tasks across professional boundaries, and improve interprofessional cooperation. However, careful consideration is required to avoid the deskilling of experienced professionals. Third, AI automation can expand flexibility by relieving clinicians from administrative duties, thereby concentrating their efforts on patient care. Remote monitoring and improved scheduling can help integrate work with other life domains. Fourth, while AI may reduce problem-solving opportunities in certain areas, it can open new pathways, particularly for nurses. Finally, task identity and variety are essential job characteristics for intrinsic motivation and worker engagement but could be compromised depending on how AI tools are designed and implemented.

**Conclusions:**

This study demonstrates AI’s capacity to mitigate stress and improve work conditions for ICU nurses and physicians, thereby contributing to resolving health care staffing shortages. AI solutions that are thoughtfully designed in line with the principles for good work design can enhance intrinsic motivation, learning, and worker well-being, thus providing strategic value for hospital management, policy makers, and health care professionals alike.

## Introduction

According to projections by the World Health Organization, by 2030, the world will witness a global deficit of 18 million health care workers, more than double the current shortfall, with potentially far-reaching implications for patient care and health care delivery [1,2]. This alarming prediction underscores not only a public health crisis but also a wider social and economic dilemma, as the shortage of health care workers significantly impairs the resilience of health systems, undermines public health outcomes, and exacerbates inequities in health care access [3]. This global concern is attributed to demographic changes such as an aging population, an increase in chronic illnesses, and a scarcity of both educational and financial resources [4,5]. Escalating this issue are high rates of absence and turnover, driven by an unprecedented level of physician and nurse burnout due to psychosocial stressors and occupational hazards such as exposure to infectious diseases, shiftwork, or ergonomic challenges, that place health care workers at risk for long-term mental and physical health consequences [6-9]. These conditions are aggravated by the emotional labor and the high-stakes environment of health care settings, resulting in professionals leaving their careers due to overwork, demotivation, or dissatisfaction [10,11].

Artificial intelligence (AI) and machine learning–based applications have emerged as promising tools to address these challenges by potentially improving work conditions and mitigating personnel shortages [12-14]. Yet, the adoption of AI in real-world health care settings has been notably sluggish, a trend associated, in part, with high acquisition costs, increased training needs, and other implementation challenges such as the “black box” nature of AI systems [15,16]. Due to the opacity of current AI systems, health care practitioners often are uncertain about the AI decision-making process, which has implications for trust and AI acceptance [15,17,18]. Moreover, it is a fallacy to presume that AI will automatically enhance human performance and work conditions. As past evidence shows, the introduction of digital technologies intended to improve the work of health care professionals has, at times, introduced new burdens on workers due to systems being ill-designed, with scant regard for the nuances of organizational and worker needs [19-23].

For technology to have the desired positive impact on performance and to engender motivating working conditions for health care staff, a comprehensive sociotechnical system design approach is required—one that incorporates principles of human-centered design along with work design [24,25]. This approach advocates for technology, organizational processes, and human tasks to be jointly designed and implemented to fully harness the benefits of a new technology such as AI [26,27].

While there is abundant research on the human-centered design of AI in health care—with a primary focus on enhancing the user experience and facilitating the interaction between single users and AI [28]—research often overlooks how multiple clinicians use AI collaboratively as an integral part of their work tasks and professional roles. Therein lies a research gap: understanding the use of AI beyond individual interactions to encompass the multiprofessional dynamic collaborations so vital to today’s complex health care delivery systems.

**Figure 1:**
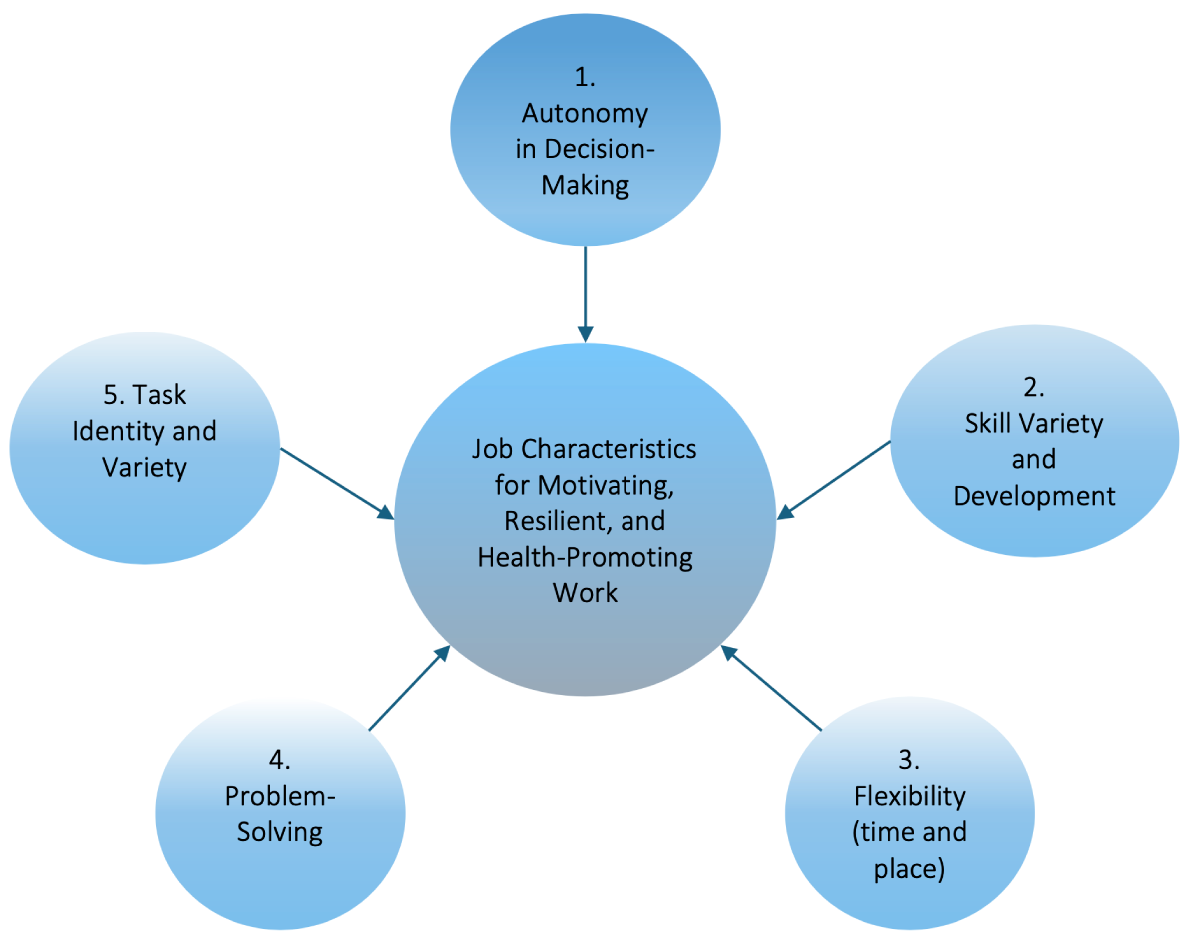
Five job characteristics for motivating, resilient, and health-promoting work.

We, therefore, examine the design of human tasks and processes within the framework of sociotechnical systems in the introduction of AI within dynamic interprofessional team collaboration in critical care settings. We apply the well-established and empirically validated work system assessment tool COMPASS (Complementary Analysis of Sociotechnical Systems [29-31]), to analyze structured data from workplace observation and interviews and to assess five job characteristics essential for enhancing intrinsic motivation, learning, and resilience among health care workers [32-34]. As depicted in Figure 1, the five job characteristics of human-centered work design are (1) autonomy in decision-making; (2) skill variety and competence development; (3) flexibility in terms of time, place, and type of work; (4) problem-solving opportunities; and (5) task identity and variety.

Our study of the sociotechnical integration of AI centers around critical care teams, specifically intensive care unit (ICU) nurses and physicians. The ICU context was selected due to the particularly high job demands and increased rates of burnout, which were exacerbated by the extreme work conditions during the SARS-CoV-2 pandemic [35-37]. Because the joint optimization of social and technical aspects of work systems must be considered before a technology is introduced into the workplace [38], we first assess the current work situation in ICUs to then compare it with future work scenarios after AI implementation. This comparison is based on the optimal levels of human-AI teaming for core ICU tasks as identified in a prior study [39] and key job characteristics for motivating, resilient, and health-promoting work. Our findings lead to actionable recommendations for AI design and implementation that alleviate working conditions for critical care nurses and physicians to ultimately improve the quality of patient care.

## Methods

### Ethical Considerations

Ethics approval was obtained from the ETH Zurich Ethics Commission (EK 2019-N-51 and EK 2019-N-190). Informed consent was obtained from participants before any form of data collection and the option to opt-out was provided. All data were anonymized. Participants did not receive any form of compensation. No patient data or information were collected.

### Study Context

The study was carried out in six ICUs that were selected based on unit size (an average of 10 to 14 beds per unit) and patient scope (a capacity of about 800 patients treated per year). Of the 6 ICUs contacted, all agreed to participate in the study. Working shifts lasted 8.5 hours (morning 7 AM to 3:30 PM, afternoon 3 PM to 11:30 PM, and night 11 PM to 7:30 AM).

### Sampling Strategy

In line with the COMPASS guidelines (Multimedia Appendix 1), we observed 5 morning shifts in each ICU, resulting in a total of 30 shifts lasting 8.5 hours each. Contingent upon each ICU’s number of beds, the team composition varied slightly, with 6 to 8 physicians and 8 to 12 nurses working together for each shift. This resulted in a total of approximately 559 health care professionals observed in this study. All nurses were registered nurses. Nurses in training and nurse practitioners were excluded from the study as they could have distorted the results based on their lower level of expertise and potentially different experience of work demands. Resident physicians oversaw up to 5 patients and attending physicians were ultimately responsible for all patients in the unit. Due to the relatively small number of attending physicians working in each unit, we combined residents and attending physicians into one professional group, hereafter referred to as physicians. To complement the worker perspectives, we additionally conduct n=12 structured interviews (6 head physicians, 5 nursing staff heads, and one head of nursing education).

### Work System Assessment

#### Assessment Tool

We used the well-established and empirically validated work system assessment tool COMPASS [29-31,40] to assess and compare the current and future work states across the 6 ICUs. COMPASS analyzes structured data from workplace observations and work unit head interviews using normative criteria based on sociotechnical systems theory (Multimedia Appendix 1).

#### Workplace Observations

The first author (NB)—a work and organizational psychologist with 15 years of experience in work system analysis and qualitative research methodology—applied the structured observational guidelines provided by COMPASS, to observe the work practices of ICU nurses and physicians in-situ (see the full set of observational guidelines and criteria in Multimedia Appendix 1). As a nonparticipant observer, she took detailed notes on a notepad standing in the background about five feet away, and where suitable, asked clarifying questions from health care professionals.

#### Interviews With Work Unit Heads

In addition to workplace observations, the first author (NB) also conducted 12 structured COMPASS interviews with each of the 6 ICU head physicians, 5 nursing staff heads, and 1 head of nursing education. Interview questions related to the overall work system structure, professional roles and responsibilities, work process variances, and disturbances (see the full list of interview questions in Multimedia Appendix 2). Interviews were conducted in single-person offices, lasted around 60 minutes, and were audio-recorded and transcribed ad verbatim.

#### Data Analysis

Interview transcripts and notes from observations were methodically analyzed using the COMPASS guidelines [29,30] and a grounded theory approach [41] to ensure qualitative rigor in qualitative research [42]. The software MAXQDA (version 2020; Verbi) was used to facilitate the analytical process [43]. Qualitative results are reported as per the standard published by the Academy of Medicine [44] (see the completed checklist in the Multimedia Appendix 3). To further enhance the trustworthiness of results, the data from observations and interviews were triangulated with additional data gathered from hospital documentation such as descriptions of job profiles and professional competencies, organizational charts, and technology user manuals. A total of 6-member checking workshops [45] were conducted (one in each unit), and 2 work unit heads validated the accuracy and adequacy of the analysis concerning the correctness of medical knowledge, contextual fit of interpretations, and adequate use of terminology [46].

#### Mixed Methods Study to Identify Optimal Levels of Human-AI Teaming Across ICU Tasks

**Figure 2:**
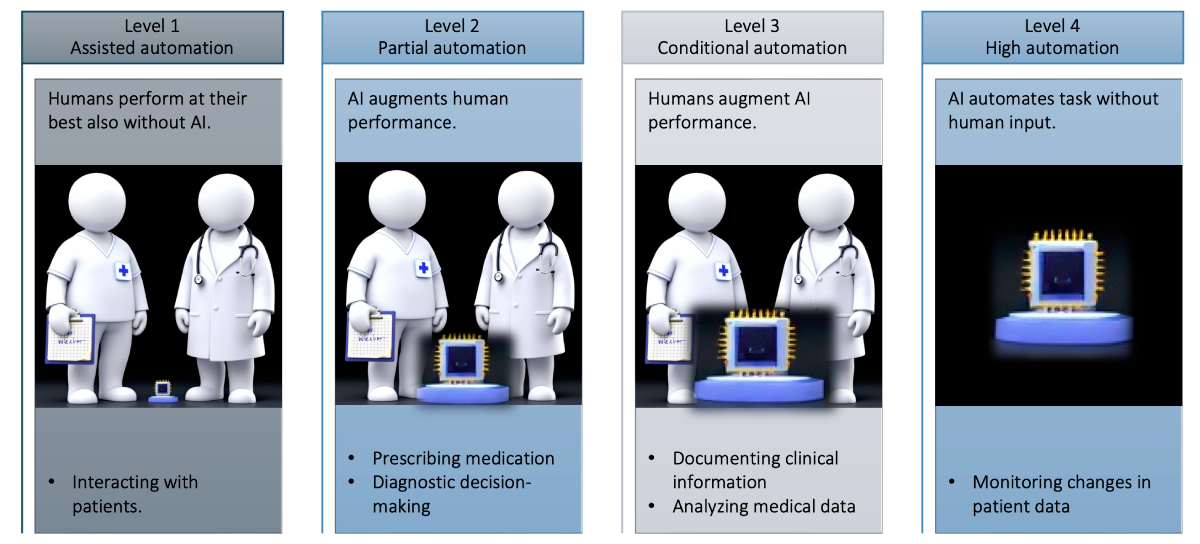
Summary of results from Bienefeld et al [39], a mixed-method study identifying optimal levels of human-AI teaming in a sample of n=19 data science experts and n=61 nurses and physicians. AI: artificial intelligence.

To assess AI-induced work system changes, we used COMPASS to evaluate each of the 5 job characteristics based on the optimal levels of human-AI teaming for each ICU task. The optimal levels of human-AI teaming for each ICU task were identified in a mixed methods study [39] and summarized in Figure 2 [39].

## Results

### Overview

In the following, we report the results of our analysis of AI-induced changes to key job characteristics for motivating, resilient, and health-promoting work in the example of ICU nurses and physicians. Table 1 summarizes the 5 job characteristics together with definitions and example quotes from interviews.

**Table 1:** Five job characteristics, their definition, and example quotes from structured interviews.

Job characteristics	Definition	Example quotes from interviews (n=12 ICU^a^ head physicians and nurses)
Autonomy in decision-making	The ability to make work-related decisions based on one’s own level of professional expertise rather than decisions by supervisors or SOPs^b^	“Of course, we need to follow lots of standards and guidelines here [in the ICU], but it doesn’t feel that way [restrictive] because every patient is different, and I have the possibility to adapt my actions to what a specific patient needs. I always need to anticipate, observe, and decide what’s next, not one patient reacts in the same way. That makes it interesting. I see the guidelines as some sort of crash barriers, they are there to protect you. But they cannot be too narrow, you need to improvise sometimes, or else it wouldn’t work. Like when things go wrong with technology, you need to solve it quickly on your own because your patient’s life depends on it. All this makes our jobs challenging but interesting.” [Head nurse, #7]
Skill variety and competence development	Opportunities for using a range of skills, applying one’s expertise and further developing competencies based on the qualification demands and the level of qualification	“Sometimes it can be tricky for us [nurses] when we are at the patient’s bedside and we clearly see that we need to do something [eg, administer some medication], but technically we can’t because it’s their [physicians’] responsibility. Rather than having to wait, it would be great if we could check with an AI or something right there in the moment and have the competence to act.” [Head of nurse education, #12]
Flexibility regarding time, place, and type of work	Influence over working conditions regarding the type and place of work and temporal flexibility regarding when the work is done	“[The lack of flexibility] in scheduling is what makes it difficult for many of us [nurses]. Especially when you have kids, [working in shifts] is just not compatible. We are quite a good team and organize ourselves via WhatsApp to swap around etc but that’s not a great solution. Or if we could at least have lunch in the canteen [away from the ICU], to air out our heads or get some air, that would already help. Sometimes, it feels like you are tethered to your patient’s bed all day long.” [Head nurse, #8].
Problem-solving opportunities	The degree of cognitive complexity required for problem-solving tasks and the possibility to perform problem-solving tasks	“Doing the mentally challenging detective work [to diagnose a patient] that’s what makes this profession [of an ICU physician] so rewarding. When you are able to make a very sick patient well again because you figure out how all the pieces of the puzzle fit together. I think that’s an important part [of being a physician] for most of us and I sure wouldn’t want to miss it.” [Head physician, #1]
Task identity and variety	The ability to perform complete tasks or processes and to perform multiple tasks within the work system	“There is never a dull moment here [in the ICU] for sure. So many different things to do, maybe sometimes too many [laughs]. To me, and I think many of my colleagues is to see when a patient comes to us, very sick and we think he [or she] might not survive. And after several weeks here [in the ICU], he [or she] can go home to his [or her] family. Then we know, we [the team of physicians and nurses] did everything we could to make this happen and that’s when I know why I like this job.” [Head physician, #3].

^a^ICU: intensive care unit.

^b^SOP: standard operating procedures.

### Autonomy in Decision-Making

The generally high levels of autonomy in decision-making and the associated ability to solve problems locally at their source were identified as major strengths in the current work system without AI. When implementing AI in safety-critical systems like the ICU, it is important to maintain high levels of human autonomy to enable agility and resilience in terms of quick reactions to fast-changing patient states, varying patient volumes, technological disturbances, and fluctuating personnel resources. If AI were to drastically increase the levels of AI-based standardization and regulation (eg, to enable robust AI performance), work processes would become more rigid, people’s decision-making autonomy would be undermined and the equilibrium between standardization and flexible action by human operators could eventually tip out of balance. In addition, people’s sense of autonomy in decision-making is essential for intrinsic motivation and job satisfaction. Physicians generally have high levels of autonomy in performing tasks, though within the typical decision-making hierarchy (eg, attending physicians must sign off decisions suggested by resident physicians). With the proposed level 2 (partial automation) autonomy in decision-making can be maintained for diagnostic decision-making and prescribing medication or treatment tasks but could potentially be threatened for the task of analyzing medical data (level 3). Without AI, nurses’ autonomy in decision-making is currently limited with a high dependency on decisions made by physicians for any task that is outside of their decision-making scope (eg, physicians must sign off before medication can be administered). The use of AI could eventually increase autonomy in decision-making for nurses, for example, by using AI to perform additional tasks such as analyzing medical data (level 3) or delivering certain types of treatment (level 2). This increase, however, would require significant changes to nurses’ education, their roles, and responsibilities, as will be discussed in more detail below.

### Skill Variety and Competence Development

A second consideration for improved work design with AI is the currently low level of cross-skilling between nurses and physicians. This hampers interprofessional collaboration and problem-solving abilities as well as their competence development. Even though without AI, nurses and physicians are regularly applying their unique professional skills and can expand their knowledge through academic or on-the-job learning, these learning opportunities are mostly offered separately within each professional group. With the assistance of increasingly reliable and robust AI, for example, to analyze medical data and document clinical information (level 3), especially nurses could broaden their skill set to increase polyvalence across professional groups. AI augmentation of these tasks would thus potentially enable more cohesive and synergetic interprofessional collaboration practices. At the same time, with AI facilitating administrative tasks, physicians could spend more time interacting with patients, which would further strengthen interpersonal skills.

### Flexibility Regarding Time, and Place of Work

The work system analysis revealed that without AI, nurses and physicians have very limited control over the type, time, or place of work. This negatively impacts their sense of personal agency at work. Low flexibility is inevitable due to shift work requiring physical presence 24/7. However, low flexibility is a major barrier to intrinsic motivation and job satisfaction because it restricts people’s sense of autonomy and mobility, as well as their ability to integrate work with other life domains [47]. Flexibility could be increased, for instance, if AI would automate the task of monitoring patient data (level 4), enabling nurses to temporarily leave the patient’s bedside without missing critical events. Moreover, with highly reliable AI to provide early warnings of upcoming complications, clinical staff could intervene long before patients enter a critical state [48,49]. This would increase their ability to plan and coordinate tasks within and across professional boundaries. In addition, if AI could assist in the documentation of clinical data and the analysis of medical data (level 3), not only would there be significant efficiency gains, but also the flexibility regarding when and where to accomplish these tasks would be increased.

### Problem-Solving Opportunities

The analysis of work in the ICU without AI revealed high levels of problem-solving opportunities for physicians and medium levels for nurses. Typical activities performed by physicians such as overseeing, planning, and deciding about patients’ diagnoses and treatments, require high levels of cognitive engagement. With AI-based task augmentation in diagnostic decision-making and prescribing medication and treatment (level 2), problem-solving opportunities for physicians are likely to remain high because the ability to validate or refute the suggestions made by an AI requires advanced expert knowledge and problem-solving skills. For nurses, a significant amount of attention and cognitive resources are needed for monitoring patients and interacting with them. With AI automation of monitoring tasks (level 4), nurses would likely require fewer cognitive resources in this area, which would free up cognitive capacity for other problem-solving tasks. For example, experienced nurses who have developed implicit knowledge or intuitive hunches about upcoming patient complications but are uncertain whether to communicate this information across interprofessional hierarchies could use AI to analyze medical data (level 3) in order to validate (or refute) these hunches, which would ultimately improve knowledge sharing and problem-solving in interprofessional care teams.

### Task Identity and Variety

The completeness of tasks (eg, doing a work task or process from start to finish), is the basis for task identity; which enables local control of variances and disturbances and allows workers to anticipate and swiftly react to upcoming problems. Without AI, task identity and variety are rated as medium for both nurses and physicians. Physicians concentrate on patient diagnosis, goal setting, and planning behaviors, while nurses focus more on preparation, execution, and controlling behaviors, as well as patient care activities. The suggested levels of AI automation for three core tasks (monitoring medical data, documenting medical information, and analyzing medical data), could lead to a significant decrease in task identity and variety. Furthermore, if nurses and physicians were to take on only supervisory control or trouble-shooting roles when collaborating with AI, their ability to solve problems locally and directly would be heavily diminished. However, whether such changes negatively impact job satisfaction and well-being depends on how important professionals perceive each task as part of their identity. For instance, (partly) automating the labor-intensive and tedious task of documenting medical information might be a welcome relief for physicians, whereas an AI-based analysis of medical data might impair their capacity to anticipate and adequately react to patient complications [50,51].

**Figure 3:**
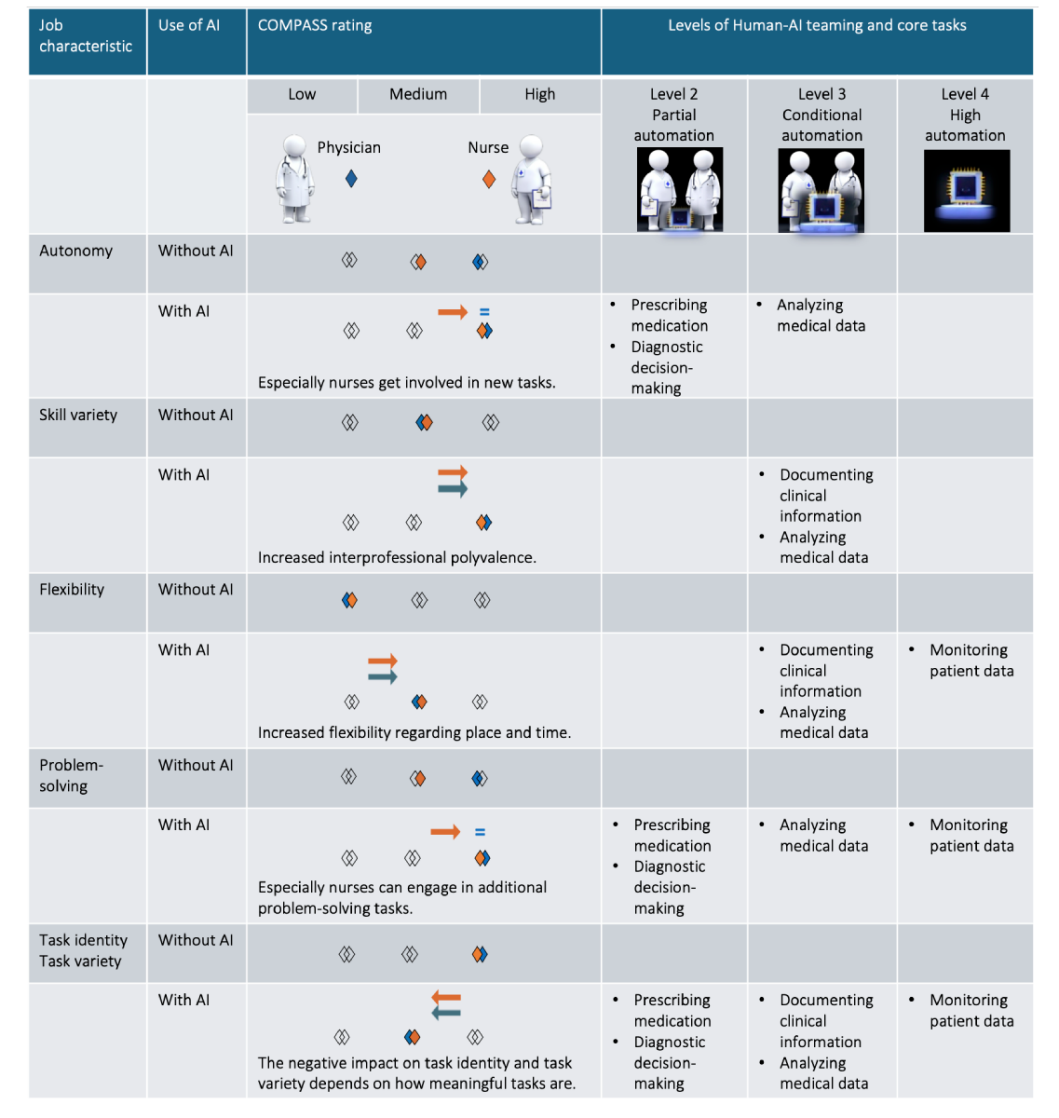
Summary of AI-induced changes to key job characteristics, based on a comparative analysis of ICU nurses’ and physicians’ work with versus without AI combining observational data (approximately 559 ICU nurses and physicians) and data from structured interviews (n=12 ICU head nurses and physicians). Arrows pointing to the right signify an improvement; arrows to the left a deterioration. The equal sign signifies no change. Nurse-specific changes are displayed in orange; physician-specific changes are in blue. The category “Nurses” includes only registered nurses. The category “Physicians” combines resident and attending physicians. AI: artificial intelligence; COMPASS: Complementary Analysis of Sociotechnical Systems; ICU: intensive care unit.

Figure 3 shows the comparative summary of results, sorted along the 5 job characteristics and optimal levels of human-AI teaming [39]. Level 1 automation is not included because AI developers and clinicians saw the use of AI in direct patient interactions as undesirable due to social and ethical concerns.

## Discussion

### Principal Findings

This study has explored how the use of AI can mitigate the increasing rates of stress and dissatisfaction among overworked ICU nurses and physicians, thus improving their work conditions and potentially contributing to reducing the global scarcity of skilled labor in this domain. We applied a sociotechnical system perspective in the analysis of current and future work scenarios to identify how AI impacts key job characteristics for motivating, resilient, and health-promoting work. As discussed in detail below, our findings highlight four areas where AI can enhance work conditions for at least one professional group and one area where the application of AI should be cautiously approached.

First, autonomy in decision-making is a fundamental aspect of work design due to its impact on multiple outcomes such as stress, motivation, learning, and performance. From a stress perspective, autonomy enables individuals to exert control over environmental demands, thereby reducing stress [52]. Moreover, extensive research suggests that job autonomy contributes to a sense of meaning and purpose in the workplace [53]. This, in turn, leads to lower turnover rates, decreased absenteeism, and increased creativity, proactivity, and, ultimately, performance [54]. Autonomy leads to improved problem-solving because decisions can be made locally, that is, problems are solved directly at the source where they arise [26,55]. A decrease in autonomy in decision-making would not only lead to the loss of these beneficial effects but likely result in limited AI acceptance and adequate use altogether. A recent study demonstrated that when radiologists felt their autonomy in decision-making was reduced by AI (because they could not understand why the system made certain recommendations), they refused to use it or found ways to override the system. Other AI systems that provided higher levels of transparency and allowed radiologists to feel “in charge” had higher acceptance rates and truly augmented their work [56]. Similar results were found in a study on an AI-based system for sepsis prediction [57] and in a study where data scientists and clinicians co-designed an algorithm to predict delayed cerebral ischemia [18]. In line with this literature and work design theory [58], maintaining human autonomy in decision-making is crucial and can be achieved by designing AI systems that provide high levels of transparency, predictability, and controllability [39].

Second, AI can be used to boost skill variety and competence development, two important job characteristics for perceiving work as stimulating, experiencing mastery of one’s work, and enabling lifelong learning [59]. Especially nurses would benefit from AI to develop new competencies and gain additional responsibilities. AI-enabled upskilling would not only be an important source of personal growth and motivation for nurses but could create a much-needed interprofessional skill set for managing clinical staff shortages. Furthermore, training nurses and physicians on how to best collaborate with AI—and each other—could help improve interprofessional collaboration overall [60]. On the other hand, failure to consider skill variety and competence development can lead to the deskilling of experienced professionals, that is, the loss of relevant knowledge and skills over time [61]. This would be problematic not only for experienced professionals but especially for younger colleagues who still need to develop a broad set of skills and expertise to be able to evaluate AI-based recommendations and intervene in case of system malfunctions.

Third, providing people with more flexibility regarding time, type, and place of work stimulates personal agency, which is at the core of intrinsic motivation [62]. AI could be used to increase flexibility by (partly) automating the tasks of monitoring patient data and documenting clinical information. By liberating nurses and physicians from such time-consuming monitoring and administrative tasks, they could engage in more meaningful work such as interacting with and more directly caring for their patients. Moving away from the computer and back to the patient’s bedside is a wish many health care professionals entertain. Strengthening the uniquely human qualities of empathy, compassion, and interpersonal connection has become increasingly important in a technology-driven world [63]. AI could also increase flexibility regarding the place of work (eg, by predictive analytics and remote patient monitoring [48,64]) and time of work (eg, by designing smart rosters that consider individual needs and preferences). Increased flexibility regarding the time and place of work would ultimately facilitate better work-life integration to accommodate family and other life domains—a known factor associated with high turnover rates in health care professionals [65,66].

Fourth, problem-solving opportunities help create stimulating and meaningful work [67,68]. According to our assessment, problem-solving opportunities are likely to stay high for physicians because they remain in control of tasks such as diagnostic decision-making. For nurses, problem-solving opportunities were associated mainly with monitoring activities. With the automation of monitoring tasks by AI, nurses would have fewer problem-solving needs in this domain, but AI could create new opportunities for problem-solving in other areas. For instance, by developing new skills and using AI to analyze medical data, nurses could engage more deeply in cognitive problem-solving activities, which would deepen their understanding of a patient’s medical condition and likely improve interprofessional communication and collaboration. However, given the current hierarchical structure of ICU teams, such changes would require significant adaptations in nurses’ educational curricula, roles, and career paths, including new regulations regarding legal accountability and reimbursement strategies [69,70].

Finally, task identity and variety are important factors for intrinsic motivation and worker engagement, and were rated as high in work conditions without AI. To maintain high task identity and variety, it is important to balance different forms of human-AI teaming as suggested by Bienefeld et al [39]. Assigning humans with leftover tasks (ie, tasks that cannot be executed by technology) can otherwise result in decreased motivation and make automated systems brittle and prone to errors [71,72]. Against the common belief that automating complex tasks leaves the easy aspects for humans, automation can make the role of humans more challenging, thus hindering their ability to intervene effectively in the event of system failures. This phenomenon, known as the “out of the loop” performance problem [73], results in human operators becoming progressively less capable of detecting system errors and reacting appropriately when automation malfunctions. This problem has been observed, for example, in the aviation industry, where pilots could no longer foresee or understand the automated actions of the flight management system, prompting inappropriate reactions that led to many tragic accidents [74-76]. Moreover, even as the potential of AI tools in health care increases and more and more tasks can potentially be automated or augmented by AI [77], it is important to allow ICU nurses and physicians to engage in a range of activities to maintain adequate levels of task variety.

Considering today’s grand health care challenges such as the high rates of attrition and shortage of skilled labor, we hope that our predictive analysis of how AI will impact the quality of work of nurses and physicians inspires all involved stakeholder groups (eg AI developers, hospital management, and policy makers) to think carefully about whether a given technology really improves the work task or process in question. Our results predict higher worker motivation, satisfaction, and well-being and fewer AI implementation hurdles if AI tools are developed and implemented in line with the principles for good work and sociotechnical system design [32,58,78]. Future researchers should continue to analyze the short and long-term effects of AI implementation in real-world health care settings, also considering additional factors such as resistance to change, team dynamics, and the role of leadership [79]. Implementation research [80] practices, like technological co-design [18] and stakeholder involvement [81], could be used to facilitate such endeavors.

### Limitations and Future Research Directions

As with any study, there are various limitations to consider when interpreting the results. While the COMPASS methodology is a theory-based, empirically validated, and highly structured tool in sociotechnical systems and work design research, we are mindful of the potential biases such as the Hawthorne effect [82] such a tool may introduce. In this study, we have undertaken due diligence to minimize such biases by taking the following measures: (1) observations were conducted as unobtrusively as possible with the observer wearing nurse gowns and standing in the background about five feet away, (2) evenly distributing observations and interviews across the six ICUs to ensure comparability, (3) standardized observer and interview guidelines (Multimedia Appendices 1 and 2), (4) extensive observer training (>15 years of experience using the COMPASS tool), and (5) centering data collection within one experienced qualitative researcher with domain expertise in work and organizational psychology to avoid interrater bias [83]. Furthermore, our findings are likely most applicable to settings with similar boundary conditions regarding workload, tasks, and team dynamics (eg, emergency or acute care teams). Nonetheless, the qualitative nature of our work has allowed us to capture the situated work practices of ICU staff, which we hope provides valuable insights for future research and real-world clinical practice.

Even though we included a relatively large sample of approximately 570 ICU nurses and physicians in this study, we may not have captured the full spectrum of experiences within interprofessional ICU teams.

Future research should examine how additional roles, particularly those with lower levels of experience (eg, nurse students and junior residents), perceive and interact with AI. The use of AI is often seen as particularly beneficial for less experienced professionals [84] but at the same time, is a cause of concern: Less experienced professionals may rely too much on the assistance of AI to perform their work, thus cannot build important skills, and lack the necessary expertise to verify the adequacy of AI outputs. Studies to examine the nuanced effects across different levels of expertise are needed to avoid these dangers and to build AI systems that support all users effectively.

Finally, despite our focus on improving job resources through AI-based technologies, ICU nurses and physicians face multiple job demands such as time pressure, very complex patient cases, and emotionally demanding situations that may continue to exert a toll on them despite AI support. As previously mentioned, a balance between job resources and job demands [33,34] can render these demands tolerable. People-focused strategies such as mentorship or emotional support programs might also help to mitigate the many challenges [85]. In addition, exploring job crafting initiatives [86,87] could enrich our understanding as a complement to the top-down approach used in this study. For instance, job crafting activities, whereby workers themselves initiate changes to their work conditions, have proven highly effective in bypassing potential management or union resistance [58].

Finally, this paper could not delve into broader issues like economic pressures in health care. While worker motivation is multifaceted, fair pay as a fundamental need is, of course, also pivotal [88].

### Conclusions

The integration of AI into critical care work presents multifaceted opportunities to enhance job satisfaction and well-being and ease the burden on staff. Specifically, our analysis demonstrates that AI can augment decision-making autonomy, encourage skill diversity and competence development, and increase flexibility—each a critical determinant of job satisfaction, well-being, and professional retention. Moreover, AI’s potential to redistribute routine and administrative tasks can allow health care professionals to allocate more time to patient care and collaborative problem-solving, thus amplifying their effectiveness and job fulfillment.

It is imperative to say that AI’s contribution extends beyond mere numerical staffing solutions. By strategically leveraging AI’s capabilities, health care systems can enhance the quality of work for ICU professionals, fostering environments where job resources are amplified and job demands are diminished. This, in turn, can lead to heightened professional engagement and a corresponding reduction in the attrition rates that exacerbate staffing shortages.

However, it is critical to acknowledge that the deployment of AI must be thoughtfully managed to prevent the deskilling of professionals and to maintain the integrity of task identity and variety, which are essential criteria for continuously safe performance. Thus, while AI holds promise for addressing aspects of the health care staffing crisis, these technologies must be used as part of a broader strategy that includes sociotechnical system design and human-centric principles.

To this end, we conclude that AI—when incorporated in alignment with good work and sociotechnical system design principles—has the potential to significantly alleviate health care staffing shortages by making the work of nurses and physicians more attractive. The study results indicate that by tailoring AI to redistribute work tasks based on the synergistic interplay between human and AI capabilities, intrinsic motivation, resilience, and worker well-being can be elevated, and the retention of professional expertise can be ensured. Future research into the sociotechnical integration of AI in health care should look to include a wider array of team-based settings beyond critical care to encompass a broader spectrum of workers and explore the long-term impacts on the health care workforce and the patients they serve.

## Appendix

Multimedia Appendix 1 COMPASS Observation Guidelines and Criteria.

Multimedia Appendix 2 COMPASS Interview Guidlines.

Multimedia Appendix 3 Completed Checklist for Qualitative Research.

Multimedia Appendix 4 Declaration of the Use of Generative AI.

## Abbreviations

AI: artificial intelligence
COMPASS: Complementary Analysis of Sociotechnical Systems
ICU: intensive care unit

## Data Availability

Datasets generated during or analyzed during this study are available in the ETH Zurich Research Collection repository and accessible by the digital object identifier (DOI; 10.3929/ethz-b-000693893). The DOI issued to datasets in the repository can be included as part of a data citation in publications, allowing the datasets underpinning a publication to be identified and accessed. The full transcripts of the interviews are not publicly available to minimize the risk of participant reidentification. Summaries of the interview contents and related metadata that support these findings are available from the corresponding author upon reasonable request.
