# Supplementary material for: AI Interventions to Alleviate Healthcare Shortages and Enhance Work Conditions in Critical Care: Qualitative Analysis
Source: J Med Internet Res. 2025 Jan 13;27:e50852. doi: 10.2196/50852 (PMC11773285; doi:10.2196/50852)
Supplement: Multimedia Appendix 1 [file jmir_v27i1e50852_app1.pdf]

COMPASS: Observation Guidelines and Criteria for the Assessment of Work Tasks

(based on Boos et al., 2013, Grote, 2000, and Waefler et al., 2003)

In the following, the five criteria for assessing individual work tasks are described. Characteristics of individual work tasks relevant for evaluating each criterion are listed and general arguments for a low versus high rating are provided. Detailed attention must be paid to documenting each work task and process. Concrete examples and multiple arguments must be collected as a comprehensive basis for the evaluation of each criterion. Criteria are assessed by listing all examples and arguments for low, medium, and high ratings and selecting the rating with the most arguments in favor:

Criterion X		
Low rating	Medium rating	High rating
Argument 1, Example 1 and 2<br>Argument 2, Example 3	Argument 3, Examples 4-6<br>Argument 4, Example 7<br>Argument 5, Examples 8 & 9<br>Argument 6, Examples 10-13	Argument 1, Example 14
Assessment = Medium		

Autonomy in decision-making

Autonomy in decision-making is characterized by the opportunity for self-determination regarding the setting of goals and making decisions based on one's professional expertise rather than having to follow decisions made by supervisors or based on rigid standard operating procedures (SOPs) and guidelines.

**Low autonomy in decision-making**	**High autonomy in decision-making**
- Almost no decisions can be made by employees.<br>- All or most decisions are made by the supervisor or people outside of the work system.<br>- All or most decisions are regulated by rigid SOPs and guidelines.	- All or most decisions can be made by employees themselves.<br>- Regular group meetings or other decision-making processes exist.<br>- Flexible guidelines allow adaptation.

Skill variety & competence development

Skill variety and competence development are rated based on the ability to apply a variety of skills in performing a range of different tasks (e.g., cooperating with different people, and following different sets of work procedures). Skill variety allows the employee to gain experience with different situations and demands and hence promotes flexible action. Opportunities for learning and competence development are determined by the (mis-) match

between qualification demands inherent to the tasks and the employee's qualification level. The criterion is a measure of the employees' opportunities to apply and to further develop their qualifications in performing their work. The focus is on the frequency of application of existing knowledge as well as on the frequency of demands for gaining new knowledge.

**Low opportunities for learning and competence development**	**High opportunities for learning and competence development**
- Repetitive tasks with identical or very similar demands require few skills.<br>- The full scope of professional qualifications is employed once a year at the most.<br>- There is no need or opportunity for additional training or competence development.	- Employees perform a broad range of tasks requiring a variety of skills.<br>- The full scope of existing skills and knowledge is used at least weekly.<br>- Additional training lasting several days is required at least once a year.

Flexibility regarding time, place, and type of work

Flexibility regarding time, place, and type of work allows workers to have some influence over working conditions. Such influence can concern choosing one’s assignments of tasks/orders, quantitative or qualitative characteristics of tasks, and general organizational issues like where and when to work and allocation of workspace. Perceived influence over working conditions is a precondition for feeling responsible and committed to one's work. Temporal flexibility describes the predictability of and influence on temporal demands, created, for instance, by externally defined deadlines and time constraints, general time pressure, and accumulation of backlogs. When employees can decide on the temporal structure of their work, they can compensate for variations in personal performance throughout the day and avoid inappropriate compression of work demands.

**Low flexibility**	**High flexibility**
- Employees have no or little influence on elements of individual or collective working conditions.<br>- Times for the beginning and end of work processes are fixed.<br>- Constant personal readiness is required to respond to changing work demands.	- Employees have significant influence on multiple elements of individual and collective working conditions.<br>- Employees can influence the beginning and end of work processes.<br>- Temporal phases of increased demands on personal readiness are predictable and can be planned.

Problem-solving opportunities

Problem-solving opportunities concern the opportunity to regularly engage in problem-solving tasks that require complex cognitive processes such as critical thinking, reflection, and abstraction.

**Low problem-solving opportunities**	**High problem-solving opportunities**
- Employees have no or very few problem-solving opportunities.<br>- Problems are handled by someone else or addressed by strict guidelines or SOPs.	- Problem solving is required frequently and handled by employees directly.<br>- Problem solving requires complex cognitive processes and critical thinking.

Task identity & variety

Task identity indicates whether the task of an individual employee incorporates goal setting, planning, preparation, executing, controlling, and correcting activities. Task identity allows employees to understand the significance of what they do and promotes their identification with the result of their work. Furthermore, task identity creates independence regarding the preceding and subsequent tasks in the flow of the work process and therefore is a basis for local control of variances and disturbances. Task variety indicates how many such tasks are performed by an individual employee.

**Low task identity & variety**	**High task identity & variety**
- The employee is involved only in one of the tasks relevant for the completion of a particular work process such as preparation, planning, executing, controlling, finishing, or maintaining.	- The employee is involved in and has control over most or all parts of a particular work process such as preparation, planning, executing, controlling, finishing, or maintaining.

References

1. Boos, D., Grote, G. & Guenter, H. A toolbox for managing organisational issues in the early stage of the development of a ubiquitous computing application. *Pers. Ubiquitous Comput.* **17**, 1261–1279 (2013).
2. Grote, G., Ryser, C., Waefler, T., Windischer, A. & Weik, S. KOMPASS: a method for complementary function allocation in automated work systems. *Int. J. Hum.-Comput. Stud.* **52**, 267–287 (2000).
3. Waefler, T., Grote, G., Windischer, A. & Ryser, C. KOMPASS: A method for complementary system design. in *Handbook of cognitive task design* 477–502 (Lawrence Erlbaum Associates Publishers, Mahwah, NJ, US, 2003). doi:10.1201/9781410607775.ch20.
