# Supplementary material for: AI Interventions to Alleviate Healthcare Shortages and Enhance Work Conditions in Critical Care: Qualitative Analysis
Source: J Med Internet Res. 2025 Jan 13;27:e50852. doi: 10.2196/50852 (PMC11773285; doi:10.2196/50852)
Supplement: Multimedia Appendix 2 [file jmir_v27i1e50852_app2.pdf]

COMPASS Interview Guidelines based on Grote (2000) and Waefler et al. (2003)

1. **Description of the work system**
   - How many people work in the work system?
   - Which other work units does the work system cooperate with?
   - What does the internal structure of the work system look like?
   - What do the overall structure of the company and the place of the work system within it look like?

2. **Tasks of the work system**
   - What are the primary tasks of the working system?
   - What are the main services provided by the work system (e.g., patient care)?
   - Besides the primary tasks, are there secondary tasks to be performed, which help to maintain or improve the work system? What are these secondary tasks?
   - What is the complete service provided (from admission to leaving the hospital)?
   - Which are the other cooperating institutions or work systems (e.g., recovery clinics, aftercare institutions)

3. **Work organization**
   - Which different functions/roles exist within the work system?
   - Which persons fulfill which functions? Are these assignments fixed or does it happen that one function gets carried out by different people?
   - Are there persons that can carry out more than one task? If so, how many people can carry out how many tasks? What are the reasons for keeping a high specialization versus supporting knowledge of different tasks?
   - Who makes what kinds of decisions?
   - Is there a clear rule on how decision-making competence is distributed?
   - Are decisions usually made by one person or collectively by work teams?
   - Who coordinates the different tasks in the work system?
   - What is the role of supervisors/team leaders in the work system?
   - How often do you (as the head of the work system, i.e., chief clinician) talk to members from other work systems? With what purpose?
   - How many hours per day/shift are you physically present in the work system?
   - Overall, does the current organization fit the purpose of fulfilling the work system's tasks? If not, what problems do you see?

4. **Communication and cooperation**
   - How much need is there to cooperate within the work system and with other work systems?
   - What are specific issues that require cooperation?
   - Which formal meetings take place in the work system?
     - Who participates in these formal meetings?
     - How frequently do such meetings take place?
     - Which topics are addressed/discussed?
   - Which informal ad hoc meetings take place in the work system?
     - Who participates in these informal meetings?
     - How frequently do such meetings take place?
     - Which topics are addressed/discussed?

- Which formal and informal ad hoc meetings take place with other work systems?
    - Who participates in these formal and informal meetings?
    - How frequently do such meetings take place?
    - Which topics are addressed/discussed?

- Beyond meetings, how does communication take place in the work system and with other work systems?

5. Work processes

- What are the main work processes carried out in the work system?
- Please describe a prototypical example of these work processes in detail:
    - Which are the preceding and subsequent work systems for this specific work process, e.g., where do the inputs for the work process come from and where do the outputs go?
    - What are the specific inputs from the preceding work system(s)?
    - What are the specific transformation processes on these inputs carried out in your work system?
    - What are the specific outputs from your work system to subsequent work system(s)?

6. Variances and disturbances

- For each input, transformation process, and output of the work process under study, ask whether variances and disturbances (could) occur, what the causes are, where the source is, and where and by whom they are discovered and handled.

7. Additional questions concerning individual tasks

- How would you describe the opportunities for learning and personal development for the employees within the work system?
- How would you describe the temporal flexibility of the employees concerning their tasks (e.g. employees can influence the beginning and the end of the work process)?
- How would you describe the employees' possibilities to influence their working conditions (e.g. quantitative or qualitative characteristics of the tasks, allocation of workspace)?

References

Grote, G., Ryser, C., Waefler, T., Windischer, A. & Weik, S. KOMPASS: a method for complementary function allocation in automated work systems. *Int. J. Hum.-Comput. Stud.* **52**, 267–287 (2000).

Waefler, T., Grote, G., Windischer, A. & Ryser, C. KOMPASS: A method for complementary system design. in *Handbook of cognitive task design* 477–502 (Lawrence Erlbaum Associates Publishers, Mahwah, NJ, US, 2003). doi:10.1201/9781410607775.ch20.
