# Supplementary material for: AI Interventions to Alleviate Healthcare Shortages and Enhance Work Conditions in Critical Care: Qualitative Analysis
Source: J Med Internet Res. 2025 Jan 13;27:e50852. doi: 10.2196/50852 (PMC11773285; doi:10.2196/50852)
Supplement: Multimedia Appendix 4 [file jmir_v27i1e50852_app4.pdf]

ChatGPT transcript of the prompt we used to generate ideas for the manuscript title and Figure 2

The following prompt was entered into the interface of the free version of ChatGPT 3.5 [1] on May 27th, 2024

Please suggest 5 different versions of an informative yet catchy title for an original paper submitted to JMIR. The title must adhere to A) the journal guidelines for titles (see link below) and B) summarize what the article is about based on the abstract provided below. Here is the link to A) https://support.jmir.org/hc/en-us/articles/115002943791-What-are-JMIR-s-guidelines-for-article-titles and here is the abstract B) Abstract Background: The escalating global scarcity of skilled healthcare professionals is a critical concern, further exacerbated by rising stress levels and clinician burnout rates. Artificial intelligence (AI) has surfaced as a potential resource to alleviate these challenges. Nevertheless, we should not assume that AI will innately augment human performance, as ill-designed healthcare technology may inadvertently impose new burdens on healthcare workers and implementation may be challenging. An in-depth understanding of how AI can effectively enhance rather than impair work conditions is therefore needed. Objective: This research investigates the efficacy of AI in alleviating stress and enriching work conditions, using Intensive Care Units (ICU) as a case study. Through a socio-technical system lens, we delineate how AI systems, tasks, and responsibilities of ICU physicians and nurses can be co-designed to foster a motivating, stimulating, and health-promoting work. Methods: We employ the socio-technical system framework COMPASS to analyze and assess five fundamental work characteristics in current and future work conditions: autonomy, skill diversity, flexibility, problem-solving opportunities, and task identity/variety. The analysis is underpinned by extensive workplace observation in six ICUs (N= 559 physicians and nurses), 12 in-depth interviews with work unit leaders, and a multi-stakeholder expert evaluation of the optimal levels of human-AI teaming. Results: The results indicate that AI holds the potential to positively impact work conditions for ICU physicians and nurses in several key areas. Firstly, autonomy is vital for stress reduction, motivation, and performance improvement. AI systems that ensure transparency, predictability, and human control can reinforce or amplify autonomy. Secondly, AI can encourage skill diversity and competence development, thus empowering clinicians to broaden their skills, increase the polyvalence of tasks, and improve interprofessional cooperation. However, careful consideration is required to avoid deskilling of experienced professionals. Thirdly, AI automation can expand flexibility by relieving clinicians from administrative duties, thereby concentrating their efforts on patient care. Remote monitoring and improved scheduling can additionally improve work-life integration. Fourthly, while AI may reduce problem-solving opportunities in certain areas, it can open new pathways, particularly for nurses. Lastly, maintaining task identity and variety is crucial to sustaining intrinsic motivation and engagement. Conclusion: The study illuminates AI's capacity to mitigate stress and improve work conditions for ICU staff, thereby contributing to resolving healthcare staffing shortages and enhancing quality of care. Embracing a socio-technical systems perspective, it advocates for AI solutions that are thoughtfully designed to enhance work motivation, satisfaction, and well-being, providing strategic value for hospital decision-makers, policy-makers, and clinicians. Keywords: artificial intelligence (AI); work design; socio-technical system; future of work; healthcare professionals; intensive care (ICU); stress mitigation.

Response:

Based on the abstract provided and JMIR’s title guidelines, here are five suggestions for an

informative yet catchy title:

"Co-Designing AI for Healthcare: Enhancing Work Conditions and Reducing Stress in Intensive Care Units"

Artificial Intelligence in ICUs: A Socio-Technical Approach to Alleviating Burnout and Improving Work Motivation"

"Leveraging AI to Improve Autonomy and Flexibility in ICUs: A Socio-Technical System Study"

"AI and Work Well-Being in Intensive Care: A Case for Enhanced Autonomy, Skill Diversity, and Flexibility"

"Optimizing AI for Healthcare Professionals: Reducing Stress and Improving Work Conditions in ICU Settings"

Each title aims to reflect the focus on AI's potential to alleviate stress and improve work conditions in ICU settings, while adhering to JMIR's preference for concise, informative titles.

The following follow-up prompt was entered into the interface of the free version of ChatGPT 3.5 [1] on May 27th, 2024

These titles are too superficial. Please generate 3 more ideas starting with the question "Can AI Mitigate Healthcare Shortages?" and putting the Analysis of work conditions to enhance job satisfaction and wellbeing in ICU physicians and nurses into the focus.

Response:

Here are three more detailed and focused title suggestions that align with your request:

"Can AI Mitigate Healthcare Shortages? Exploring the Impact of AI on Job Satisfaction and Well-Being Among ICU Healthcare Professionals"

"Can AI Mitigate Healthcare Shortages? A Socio-Technical Evaluation of AI's Role in Enhancing Work Conditions and Well-Being in ICU Physicians and Nurses"

"Can AI Mitigate Healthcare Shortages? A Systematic Analysis of Future Work Conditions to Enhance Job Satisfaction and Wellbeing in ICUs"

These titles emphasize both the potential of AI to address healthcare staffing challenges and the detailed analysis of work conditions, aiming to improve job satisfaction and well-being for ICU staff.

The following prompt was entered into the interface of the free version of ChatGPT 3.5 [1] on October 8th, 2024

I chose this title "Can AI Mitigate Healthcare Shortages? A Systematic Analysis of Future Work Conditions to Enhance Job Satisfaction and Wellbeing in ICU Physicians and Nurses". Please give

me five more versions of a title to better bring forward the qualitative nature of the research in the title per the following guidelines = Concise description of the nature and topic of the study Identifying the study as qualitative or indicating the approach (e.g., ethnography, grounded theory) or data collection methods (e.g., interview, focus group) is recommended).

Response:

Here are five versions of the title that emphasize the qualitative nature of the research, following the guidelines:

"Can AI Mitigate Healthcare Shortages? A Qualitative Study of ICU Work Conditions to Improve Job Satisfaction and Wellbeing"

"Can AI Mitigate Healthcare Shortages? An Interview-Based Qualitative Analysis of Future Work Conditions for ICU Staff"

"Can AI Mitigate Healthcare Shortages? A Socio-Technical, Qualitative Evaluation of ICU Work Conditions Through Observations and Interviews"

"Can AI Mitigate Healthcare Shortages? A Qualitative Exploration of ICU Physicians' and Nurses' Work Satisfaction and Wellbeing"

"Can AI Mitigate Healthcare Shortages? An In-Depth Qualitative Study of ICU Work Conditions Using Observations and Expert Interviews"

These versions incorporate a clear indication of qualitative methods (e.g., interviews, observations) to align with the recommended structure.

Note: The author team was inspired by these title suggestions but ultimately defined the title as follows:
Can AI Mitigate Healthcare Shortages? A Qualitative Analysis of Current and Future Work Conditions to Enhance Job Satisfaction and Wellbeing in Critical Care Nurses and Physicians

---

The following prompt was entered into the interface of the free version of GPT 4 [1] on November 29th, 2024

Create an image by drawing the following two figures side by side in the same style: Figure 1: Draw a simple, rounded human figure with no hair and an empty face to ensure a gender-neutral appearance. One figure wears scrubs-like clothing. Scrubs typically have a simple, loose-fitting design with a medical badge on the chest area. The first figure holds a clipboard. For the second figure use the same simple, rounded human figure with no hair and an empty face for consistency. Clothing: Dress the second figure in a lab coat without any gender-specific details. Stethoscope: Draw a stethoscope around the neck of the second figure. Detailed Description: Shape and Form: Both figures should be identical in shape, size, and style. Use rounded lines to create a friendly, approachable and modern appearance. Face: Keep the face empty, with no features like eyes, nose, or mouth to avoid any gender-specific

characteristics. In the middle of the two figures draw a small microchip. Same style.

Response:

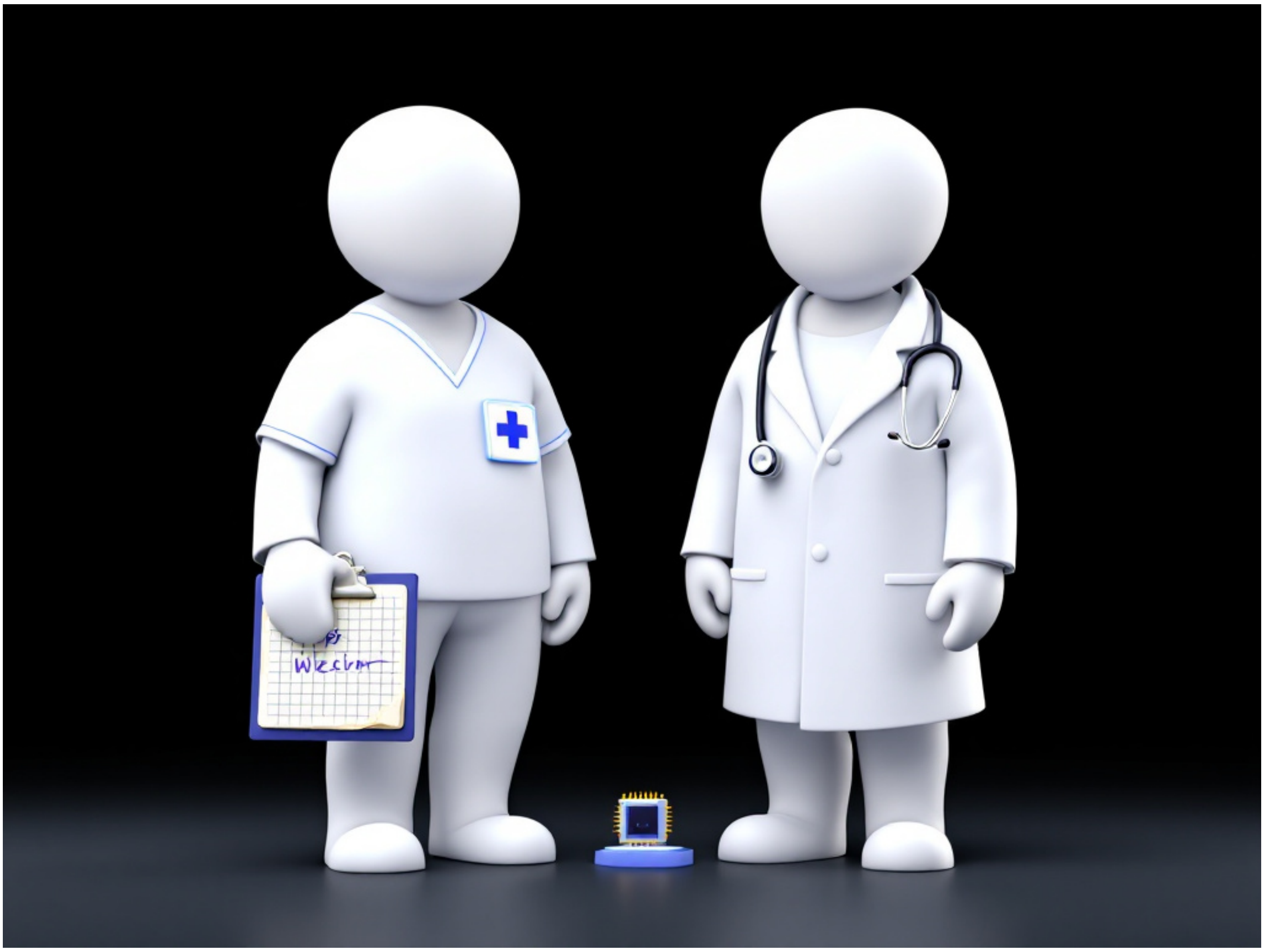

References

[1] OpenAI, *ChatGPT*. (2024). [[Large language model]]. Available: https://chat.openai.com/chat
